# Analgesics Induce Alterations in the Expression of SARS-CoV-2 Entry and Arachidonic-Acid-Metabolizing Genes in the Mouse Lungs

**DOI:** 10.3390/ph15060696

**Published:** 2022-06-01

**Authors:** Fatima Khirfan, Yazun Jarrar, Tariq Al-Qirim, Khang Wen Goh, Qais Jarrar, Chrismawan Ardianto, Mohammad Awad, Hamzeh J. Al-Ameer, Wajdy Al-Awaida, Said Moshawih, Long Chiau Ming

**Affiliations:** 1 Department of Pharmacy, Al-Zaytoonah University of Jordan, Amman 11731, Jordan; fatimakhirfan@yahoo.com (F.K.); tariq.qirim@zuj.edu.jo (T.A.-Q.); mohammadkawad96@gmail.com (M.A.); 2 Faculty of Data Science and Information Technology, INTI International University, Nilai 71800, Malaysia; khangwen.goh@newinti.edu.my; 3 Department of Applied Pharmaceutical Sciences, Faculty of Pharmacy, Al-Isra University, Amman 11622, Jordan; qais.jarrar@iu.edu.jo; 4 Department of Pharmacy Practice, Faculty of Pharmacy, Universitas Airlangga, Surabaya 60115, Indonesia; 5 Department of Biology and Biotechnology, American University of Madaba, Madaba 17110, Jordan; hamzeh_uj@yahoo.com (H.J.A.-A.); w.alawaida@aum.edu.jo (W.A.-A.); 6 PAP Rashidah Sa’adatul Bolkiah Institute of Health Sciences, Universiti Brunei Darussalam, Gadong BE1410, Brunei Darussalam; saeedmomo@hotmail.com

**Keywords:** COVID-19, lung disease, chronic respiratory disease, acute respiratory distress syndrome, gene expression

## Abstract

Paracetamol and nonsteroidal anti-inflammatory drugs are widely used in the management of respiratory viral infections. This study aimed to determine the effects of the most commonly used analgesics (paracetamol, ibuprofen, and diclofenac) on the mRNA expression of severe acute respiratory syndrome coronavirus 2 (SARS-CoV-2) entry and arachidonic-acid-metabolizing genes in mouse lungs. A total of twenty eight Balb/c mice were divided into four groups and treated separately with vehicle, paracetamol, ibuprofen, and diclofenac in clinically equivalent doses for 14 days. Then, the expressions of SARS-CoV-2 entry, *ACE2*, *TMPRSS2*, and *Ctsl* genes, in addition to the arachidonic-acid-metabolizing *cyp450*, *cox*, and *alox* genes, were analyzed using real-time PCR. Paracetamol increased the expressions of *TMPRSS2* and *Ctsl* genes by 8.5 and 5.6 folds, respectively, while ibuprofen and diclofenac significantly decreased the expression of the *ACE2* gene by more than 2.5 folds. In addition, all tested drugs downregulated (*p* < 0.05) *cox2* gene expression, and paracetamol reduced the mRNA levels of *cyp4a12* and *2j5*. These molecular alterations in diclofenac and ibuprofen were associated with pathohistological alterations, where both analgesics induced the infiltration of inflammatory cells and airway wall thickening. It is concluded that analgesics such as paracetamol, ibuprofen, and diclofenac alter the expression of SARS-CoV-2 entry and arachidonic-acid-metabolizing genes in mouse lungs.

## 1. Introduction

COVID-19 infection is caused by severe acute respiratory syndrome coronavirus 2 (SARS-CoV-2). This viral infection became a global endemic disease in 2019. The SARS-CoV-2 virus infects mainly the respiratory system and enters epithelial cells through interaction between the viral spike and specific proteins on the host cells [1,2]. SARS-CoV-2 attaches to the angiotensin-converting enzyme type 2 (ACE2) receptor at the top of host cells [3,4]. This mechanism cannot be completed without a human cathepsin L (CTSL) protease that cuts the S-glycoprotein at exact locations to let the SARS-CoV-2 attach to the host cell surface [5]. In addition, the human transmembrane protease serine 2 (TMPRSS2) stimulates the entrance of the SARS-CoV-2 virus into the epithelial cells [5,6]. TMPRSS2 works by splitting the virus, leading to the detaching of the spike piece, and helps in the spread of the SARS-CoV-2 virus [7]. It was suggested that factors that alter the expression of SARS-CoV-2 entry protein can affect the risk and severity of COVID-19 infection. 

The major symptoms of the COVID-19 infection are fever and malaise [8,9]. Therefore, analgesics and antipyretics, such as nonsteroidal anti-inflammatory drugs (NSAIDs) and paracetamol, are used to manage fever [10]. Furthermore, these analgesics can alleviate some COVID-19 infection symptoms, such as body pain [11]. NSAIDs exert their interaction through the inhibition of arachidonic acid metabolism to prostaglandins. NSAIDs can affect arachidonic-acid-metabolizing cytochrome P450s (cyp450s), cyclooxygenases (coxs), and lipooxygenases (aloxs) in different organs, including the liver, kidneys, and heart [12,13]. 

Clinical observations showed that when some patients with COVID-19 infection and no other diseases were given NSAIDs, their symptoms worsened. Additionally, another clinical study reported COVID-19 exacerbation after taking NSAIDS [14].

There is a lack of in vivo studies regarding the influence of NSAIDs and paracetamol on the expression of SARS-CoV-2 entry genes. We hypothesized that NSAIDs induced the expression of pulmonary SARS-CoV-2 entry genes. Therefore, we aimed to determine the effects of paracetamol and the most commonly used NSAIDs, ibuprofen and diclofenac, on the expression of the SARS-CoV-2 entry gene in the lungs of treated mice. Furthermore, disturbances in the expressions of mouse arachidonic-acid-metabolizing *cyp450*, *cox*, and *alox* genes caused by the analgesics in the lungs were determined.

## 2. Results

### 2.1. Physical Observation 

**Figure 1:**
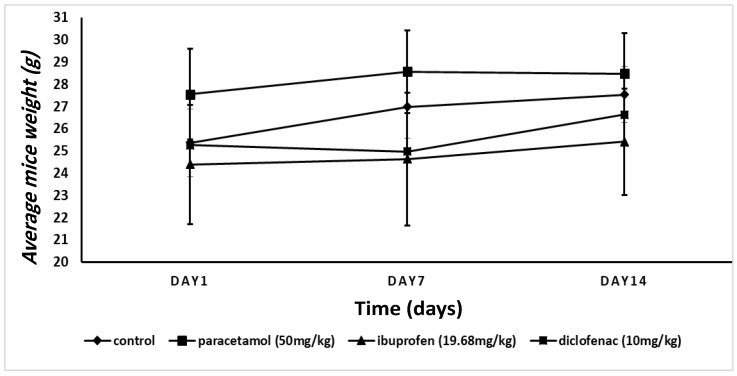
The changes in total body weight of the experimental mice. There was no significant change (*p* > 0.05, two-way ANOVA) in the total body weight after 14 days of analgesic treatment.

Figure 1 shows the change in mice weight of all tested groups. We did not find a significant change (*p* > 0.05) in their weight on the seventh or the last day of drug administration. 

### 2.2. Histological Analysis

**Figure 2:**
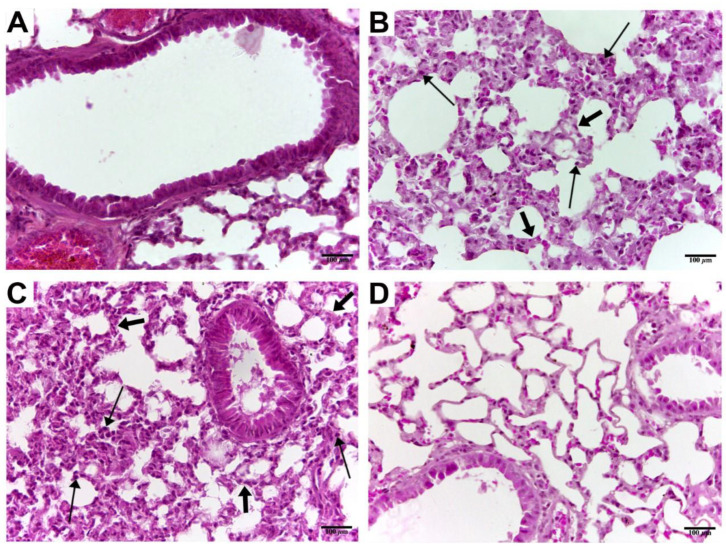
Histopathologic lungs analysis of animals after treatment. (**A**) Control lung section shows the normal structure of the bronchiole and adjacent alveoli. (**B**) Ibuprofen-treated mice representative lung section showing normal lung histology. (**C**) Diclofenac-treated mice lung tissue section showing normal bronchus and adjacent alveoli. (**D**) Paracetamol-treated mice lung tissue showing normal bronchial and alveolar tissues. Thick arrows indicate thickening in the alveolar wall; thin arrows indicate inflammatory cell infiltration. Tissue sections were stained with hematoxylin and eosin (scale bar 100 µm) and photographed at 40× magnification.

Figure 2 presents the histological sections of the mouse lungs after administration of paracetamol, ibuprofen, and diclofenac for 14 consecutive days. We found that 14 injections of ibuprofen (Figure 2B), and diclofenac (Figure 2C), but not paracetamol (Figure 2D), induced the inflammatory cell infiltration and airway wall thickening in the lungs of the treated mice.

### 2.3. mRNA Levels of SARS-CoV-2 Entry Gene 

**Figure 3:**
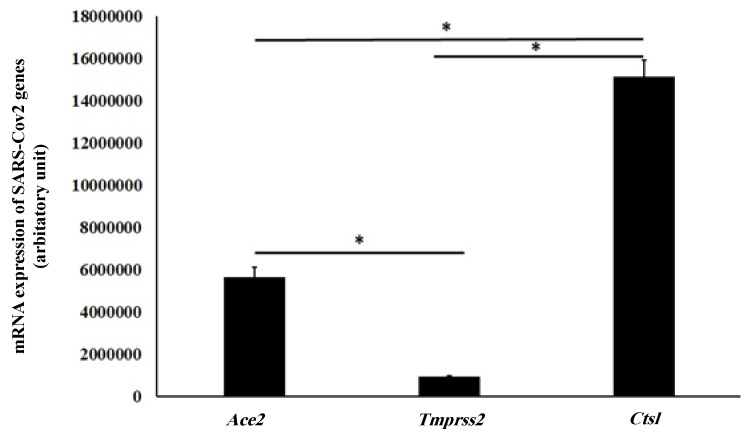
Relative mRNA expression of SARS-CoV-2 entry genes in the mouse lungs. * indicates a statistical alteration (*p* <0.05, one-way ANOVA test).

We found in this study that *Ctsl* was the most highly expressed SARS-CoV-2 entry gene in the mouse lungs. *Ctsl* was expressed 2.7 times more than *ACE2* (*p* = 0.01), which was 15 times higher than *TMPRSS2* gene (*p* < 0.0001). The relative expression of SARS-CoV-2 entry genes in the mouse lungs is illustrated in Figure 3.

**Figure 4:**
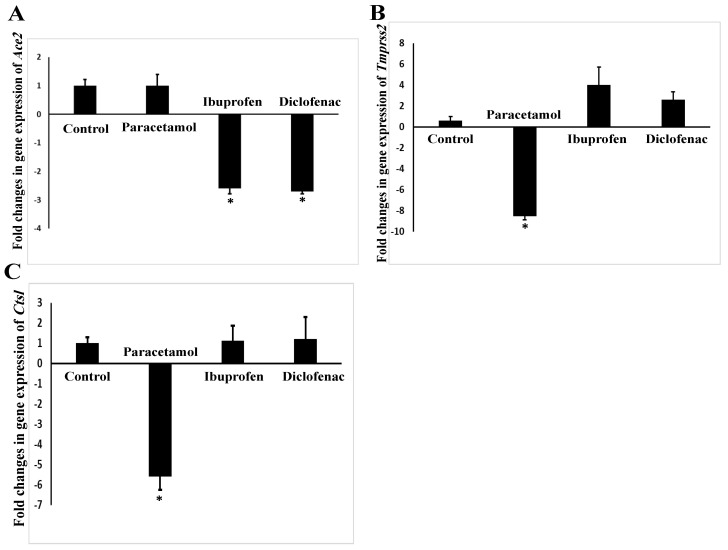
Expressions of SARS-CoV-2 entry genes *ACE2* (**A**), *TMPRSS2* (**B**), and *Ctsl* (**C**) in the lungs of NSAID- and paracetamol-treated mice. The mRNA expression of the targeted genes was quantified relative to *Actin* expression. Fold change indicates the ratio of mean expression of the NSAID- and paracetamol-treated to the control value. Negative values indicate a reduction in fold change. * indicates a statistical difference (*p* < 0.05, one-way ANOVA test) in comparison with the control group.

The mRNA expression of the *ACE2* gene was significantly downregulated in the mouse lungs after administration of ibuprofen (*p* = 0.02) and diclofenac (*p* = 0.02) by 2.6 and 2.7 folds, respectively (Figure 4A). The expression of the mouse *TMPRSS2* gene was significantly decreased (*p* = 0.008) after paracetamol treatment by 8.54 folds. Although the NSAIDs ibuprofen and diclofenac upregulated the expression of *TMPRSS2* gene by 4 and 2.6 folds, respectively (Figure 4B), this upregulation of *TMPRSS2* by NSAIDs failed to reach statistical significance (*p* = 0.10–0.27). In addition, paracetamol only downregulated the expression of the *Ctsl* gene by 5.59-fold (*p* = 0.006) (Figure 4C).

### 2.4. mRNA Levels of Arachidonic-Acid-Metabolizing cox Gene 

**Figure 5:**
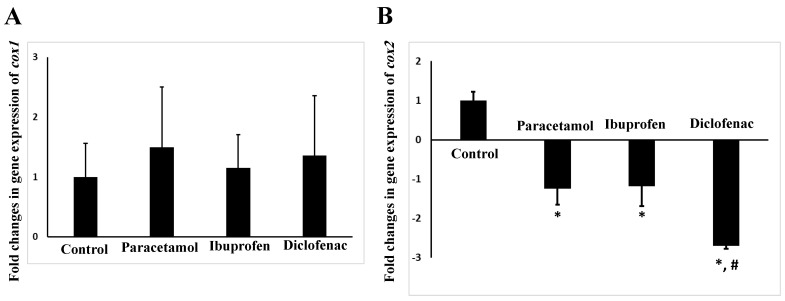
Expression of *cox1* (**A**) and *cox2* (**B**) genes in the lungs of NSAID- and paracetamol-treated mice. The target expression was quantified relative to the expression of *Actin* gene. Fold change is the ratio of mean expression of the NSAID- and paracetamol-treated to the control value. Negative values indicate a reduction in fold change. * indicates a statistical difference (*p* < 0.05, one-way ANOVA test) in comparison with the control group, while # indicates a statistical difference in comparison of diclofenac with other analgesics.

We found that all tested analgesics did not significantly affect (*p* = 0.7–0.9) the expression of the lung *cox1* gene (Figure 5A). However, all tested analgesics caused a significant (*p* = 0.01–0.04) downregulation of the mouse lung *cox2* after 14 days of administration (Figure 5B). The strongest effect on the *cox2* gene expression was observed for diclofenac, which downregulated the mRNA expression of the *cox2* gene by 2.7 folds (*p* = 0.01).

### 2.5. mRNA Levels of Arachidonic-Acid-Metabolizing alox Gene

**Figure 6:**
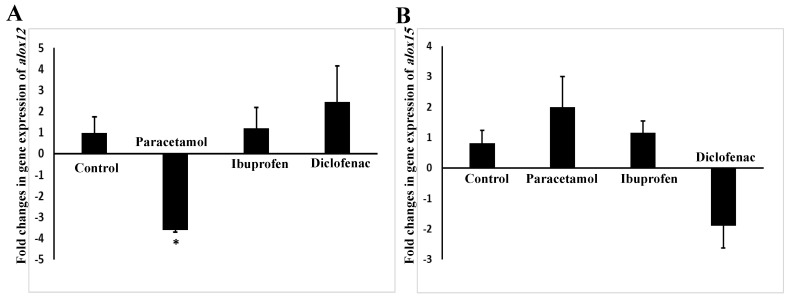
Expression of *alox12* (**A**) and 15 (**B**) genes in the lungs of NSAID- and paracetamol-treated mice. The target expression was quantified relative to the expression of actin gene. Fold change is the ratio of mean expression of the NSAID- and paracetamol-treated to the control value. Negative values indicate a reduction in fold change; * indicates a statistical difference (*p* < 0.05, one-way ANOVA test) in comparison with the control group.

We found that only paracetamol had a significant (*p* = 0.009) effect on the expression of *alox12*, as shown in Figure 6A,B. Paracetamol downregulated the expression of the *alox12* gene by 3.59 folds (Figure 6A).

### 2.6. mRNA Levels of Arachidonic-Acid-Metabolizing cyp450 Gene

**Figure 7:**
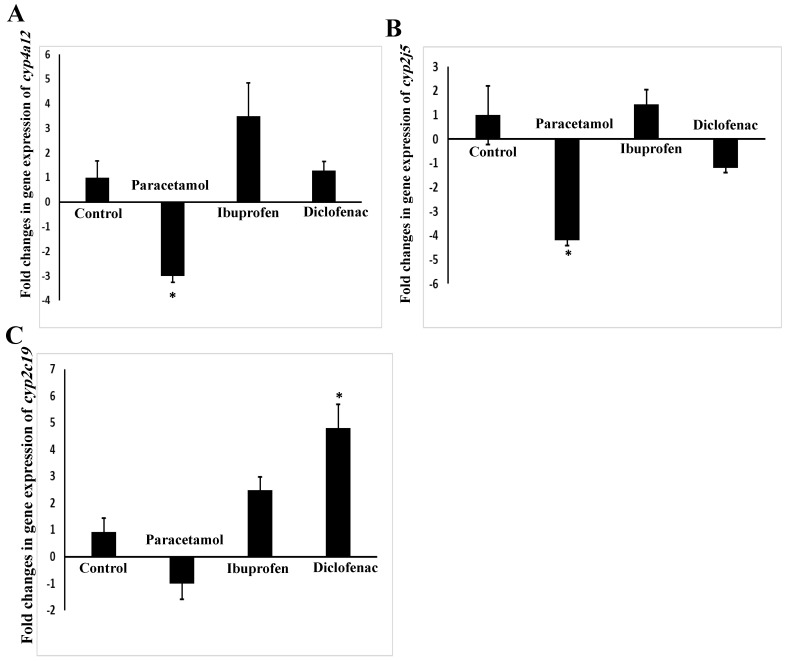
Expressions of *cyp4a12* (**A**), *cyp2j5* (**B**), and *cyp2c29* (**C**) genes in the lungs of NSAID- and paracetamol-treated mice. The target expression was quantified relative to the expression of the actin gene. Fold change is the ratio of mean expression of the NSAID- and paracetamol-treated to the control value; negative values indicate a reduction in fold change; * indicates a statistical difference (*p* < 0.05, *one-way ANOVA* test) in comparison with the control group.

Figure 7A–C presents the influences of paracetamol, ibuprofen, and diclofenac on the expression of the arachidonic-acid-metabolizing *cyp450* genes in the lungs of the treated mice. Paracetamol significantly (*p* = 0.03) downregulated the *cyp4a12* gene by three folds (Figure 7A). Additionally, paracetamol significantly reduced (*p* = 0.01) the expression of the mouse *cyp2j5* gene in the lung by 4.2 folds (Figure 7B).

Regarding *cyp2c29* gene expression, only diclofenac significantly induced (*p* = 0.009) its mRNA expression, by 4.8 folds (Figure 7C). Although the other NSAID, ibuprofen, showed a slight increase in the mRNA levels of the *cyp2c29* gene (2.3 folds), this induction failed to reach statistical significance (*p* = 0.27). Lastly, we found that the *cyp3a11* gene was not expressed in the mouse lung when tested using a RT-PCR assay.

## 3. Discussion

Analgesics are widely used in the management of fatigue and fever, which are the symptoms of viral infections, including COVID-19. However, there are controversial reports regarding the use of NSAIDs in the management of COVID-19 symptoms, and it is recommended to replace NSAIDs with paracetamol [15,16]. In this study, we showed that NSAIDs and paracetamol significantly affected the mRNA expression of the SARS-CoV-2 entry gene, and caused an imbalance in the mRNA expression of arachidonic-acid-metabolizing genes. The pattern of paracetamol’s effect on the expression of SARS-CoV-2 entry and arachidonic-acid-metabolizing genes was different than that of ibuprofen and diclofenac. These differences between paracetamol and NSAIDs, regarding their influence on the mRNA expression of SARS-CoV-2 entry genes, may explain, at least partly, the differences between paracetamol and NSAIDs in the clinical management of COVID-19 symptoms. Further clinical studies are needed to confirm the findings of this study. 

Toxicological studies used body weight and pathohistological examinations as markers of drug-induced toxicity on the animals and organs [16]. In this study, we found that 14 days of treatment with all drugs did not change the body weight. However, ibuprofen and diclofenac caused toxicological changes, as indicated by the results of the histological examination of the mouse lungs, where both NSAIDs caused infiltration of inflammatory cells and increased the thickness of the wall of the bronchioles. It was reported that NSAIDs have the capacity to induce oxidative stress in the cells [16]. Accordingly, the molecular alterations in the mRNA expression of arachidonic-acid-metabolizing genes, *Ctsl, TMPRSS2*, and *ACE2*, were associated with the toxicological effects of NSAIDs on the mouse lung *TMPRSS2ACE2.* Our results showed that paracetamol did not induce pathohistological alterations in the mouse lungs after 14 days of administration. We concluded that paracetamol is relatively safer than NSAIDs on the lungs, and that these findings support the use of paracetamol, rather than NSAIDs, for patients suffering from pulmonary diseases. 

Our findings indicated that *Ctsl* is the most highly expressed SARS-CoV-2 entry gene in the mouse lungs. This indicated that the Ctsl protein can be considered as an important target for the prevention of SARS-CoV-2 entry into epithelial lung cells. It was reported that the inhibition of Ctsl prevents severe respiratory infections caused by viral infections [17]. 

Although the *TMPRSS2* gene is expressed in lower amounts than *ACE2* and *Ctsl*, inhibitors of TMPRSS2 protein, such as ambroxol, can clinically reduce the severity of SARS-CoV infections [18]. In this study, we found that paracetamol downregulated the mRNA expression of the *Ctsl* and *TMPRSS2* genes. Our findings are in line with those of Sharif-Askari et al.: paracetamol can reduce the expression of the human *TMPRSS2* gene using in silico methods [19]. This finding indicated that paracetamol has a favorable effect over ibuprofen and diclofenac in decreasing the entry of SARS-CoV-2 into the epithelial cells. Interestingly, it was found that paracetamol has an antiviral effect [20], and that decreasing *TMPRSS2* and *Ctsl* expressions might be a mechanism of paracetamol against the entry of viruses into the host cells.

There is a controversial report regarding the correlation between human *ACE2* expression and the severity of COVID-19 infection. It was suggested that patients with increased susceptibility to COVID-19 complications have reduced levels of human ACE2 [18]. The current study reported that NSAIDs, but not paracetamol, decreased the mRNA expression of the mouse *ACE2* gene in the lungs. In agreement with the findings reported by Sharif-Askari et al., ibuprofen can reduce the expression of the human *ACE2* gene using in silico methods [21]. This finding may explain, at least in part, the harmful effects of NSAIDs on COVID-19 patients.

In this study, we found that all tested analgesics decreased the expression of the mouse *cox2* gene, which plays a major role in inflammation [22]. Therefore, this finding may explain the anti-inflammatory effect of all tested analgesics, including paracetamol [23]. Furthermore, we found that diclofenac decreased the expression of the *cox2* gene more than ibuprofen and paracetamol. Interestingly, diclofenac is considered a stronger analgesic than ibuprofen and paracetamol [24,25].

Alox12 causes bronchoconstriction by producing leukotrienes [26,27]. Some bronchodilator drugs target the formation of leukotrienes, and hence can be used in the treatment of asthma and chronic obstructive pulmonary diseases [28]. It was reported that NSAIDs are contraindicated in patients with asthma disease because that NSAIDs increase the synthesis of leukotrienes [29]. On the other hand, paracetamol is considered safer than NSAIDs as an analgesic and antipyretic for asthmatic patients [30]. We found that paracetamol decreased the mRNA expression of the mouse *alox12* gene. Therefore, we postulated that paracetamol can decrease the synthesis of leukotrienes through the downregulation of the expression of the *alox12* gene, which may explain the safety of paracetamol use among COVID-19 patients [31].

20-Hydroxyecostarionic acid (20-HETE) is synthesized by mouse cyp4a12. It was noticed that 20-HETE is overexpressed in hypoxia and vasoconstrictive pulmonary diseases [32,33]. Arachidonic acid is metabolized by mouse cyp2j5 and cyp2c19 to epoxyeicosatrienoic acids (EETs), which cause pulmonary vasoconstriction and hypoxia [33]. Paracetamol, but not NSAIDs, significantly downregulated the expression of *cyp4a12* and *cyp2j5* genes in the mouse lungs. On the other hand, we found that the *cyp2c29* gene expression was induced by the administration of diclofenac. Collectively, paracetamol decreased the mRNA expression of arachidonic-acid-metabolizing cyp450s.

In addition to arachidonic acid metabolism, *cyp2c29* is a phase I drug-metabolizing enzyme, which metabolizes many drugs, including warfarin [12]. Induction of the pulmonary cyp2c29 enzyme by diclofenac may result in an increased drug metabolism in the lung. It was reported that diclofenac decreased the expression of the hepatic *cyp2c29* gene, which was associated with hepatotoxicological alterations in the liver [34]. This indicated that diclofenac has a tissue-dependent effect on the mRNA expression of the *cyp2c29* gene [34].

This study, for the first time, revealed that analgesics such as paracetamol, ibuprofen, and diclofenac induced alterations in the expressions of the ACE2 receptor, *Ctsl*, TMPRSS2, and arachidonic-acid-metabolizing genes in mouse lungs. However, this study also had some limitations. For example, the in vivo model employed Balb/c mice, which were not infected with SARS-CoV-2. However, our focus was the molecular effects of NSAIDs and paracetamol on the mouse ACE2, TMPRSS2, and Ctsl genes, which have nucleic and amino acid sequences that are close to those of humans. Another limitation is that we did not analyze the protein expression to confirm mRNA results. Furthermore, we did not analyze the levels and concentrations of arachidonic acid metabolites, which can indicate the influence of analgesics on the production of arachidonic acid metabolites in the lungs. To verify our findings, we need more in vivo experiments on humanized ACE2 and TMPRSS2 mice. Additionally, further clinical studies are needed to confirm the findings of this study.

## 4. Material and Methods

### 4.1. Chemicals

Diclofenac sodium, ibuprofen, paracetamol, isopropyl alcohol, PEG400, and 75% alcohol were obtained from Sigma-Aldrich (St. Louis, MO, USA). Diclofenac sodium salt was solubilized in PEG400. TRIzol solution and a cDNA synthesis kit were purchased from ZYMO RESEARCH (Irvine, CA, USA). TB Green^®^ Fast qPCR Mix was purchased from Takara Bio (Kusatsu, Japan). The oligonucleotides, for PCR reaction, were bought from Integrated DNA technologies (Coralville, IA, USA).

### 4.2. Experimental Animals

Twenty eight male Balb/c mice (Mus musculus) of the same age and weight were collected from the animal house of Jordan’s Al-Zaytoonah University (Amman, Jordan). The mice were handled according to the Canadian Council on Animal Care’s guidelines [35], and the study methodology was approved by Jordan’s Al-Zaytoonah University’s ethical committee with a reference number of 04/07/2020-2021. The mice were kept at a temperature of 23 ± 1 °C with a 12 h light/12 h dark cycle. All mice were fed ad libitum with standard laboratory animal diet pellets.

### 4.3. Experimental Protocol

After a 7-day stabilization period, the twenty eight mice were divided into four groups with seven each, as follows:

(1) Control group: the mice received a once-daily intraperitoneal dose of 50% polyethylene glycol 400, the vehicle used for the solubilization of analgesic drugs.
(2) Paracetamol group: the mice were administered a once-daily intraperitoneal injection of 50 mg/kg paracetamol.
(3) Ibuprofen group: the mice were administered a once-daily intraperitoneal injection of 19.68 mg/kg ibuprofen.
(4) Diclofenac group: the mice were treated with a once-daily intraperitoneal injection of 10 mg/kg diclofenac.

The drugs were administered to the animals for a continuous 14 days. The used doses of NSAIDs corresponded to the daily dose for humans, which depend on the surface area of the animal body [12]. This period of analgesic treatment mimicked the period of disease symptoms that were used in humans and found to be able to alter the expression of arachidonic-acid-metabolizing enzyme genes [12,36]. The mice were euthanized by cervical dislocation, as suggested by the Canadian Council on Animal Care [35]. 

### 4.4. Physical Observation 

Throughout the investigation, the mice’s weights were measured three times. The first weight measurement was taken on the first day of drug administration, the second was taken one week later, and the final was taken on the last day of drug administration.

### 4.5. Histological Analysis

The histological investigation was carried out according to the previously described protocol [12]. After the mice were sacrificed, the lung samples were dissected and cleaned with 0.9% normal saline before being fixed in 10% formalin for more than one day. The samples were next dehydrated by putting them through a graded series of alcohol, followed by xylene. The lung tissues were then embedded in paraffin wax. Hematoxylin and eosin were used to stain the obtained lung sections. Lastly, a Leica^®^ (Wetzlar, Germany) microscope attached to a digital camera was used to photograph the prepared sections.

### 4.6. RNA Extraction and cDNA Synthesis

After mouse sacrifice, about 200 mg of lung was isolated from each mouse. Then, triazol, isopropyl alcohol, and 75% alcohol were used to extract the pulmonary RNA, according to the manufacturer’s instructions. Next, a cDNA Synthesis Kit^®^ was used to convert the extracted mRNA to cDNA. Briefly, 1 mg of RNA was added to a reaction mixture containing 100 pmol oligo deoxythymine, 2.5 mM dNTP, 0.1 M DTT, 1 × reverse transcriptase buffer, and 100 units of Moloney Murine Leukemia Virus reverse transcriptase, and incubated for 60 min at 37 °C. 

### 4.7. Gene Expression Analysis

The expressions of mouse *ACE2*, *Ctsl*, *TMPRSS2*, *cox1*, *cox2*, *lox12*, *lox15*, *cyp4a12*, *cyp2j5*, *cyp2c29*, and *cyp1a1* genes were examined in this research. Table 1 shows the oligonucleotides sequence, amplicon size, and annealing temperature for each amplified gene. The expressions of these targeted genes were analyzed using quantitative real-time polymerase chain reaction (qRT-PCR), as prescribed previously [37]. Briefly, 10 ng of the synthesized cDNA was added to a reaction mixture containing qPCR Master Mix and 10 pmol of forward and reverse oligonucleotides. The PCR conditions used were as follows: denaturation at 95 °C for 3 min was followed by 40 cycles of denaturation at 95 °C for 10 s and annealing at 53–58 °C for 30 s (Table 1). The mouse *Actin* gene was used as a housekeeping gene in this study, and the expression of the genes was calculated using the ^ΔΔ^CT method [38].

### 4.8. Statistical Analysis

The change in the mRNA expression of the examined genes following analgesics administration is expressed as a number of fold changes in comparison with the control group. The mRNA expression of the tested genes, in each group, was normally distributed according to the Kolmogorov–Smirnov test. The comparison between the control and other groups was carried out using a two-way (for the body weight) and one-way (for the gene expression) analysis of variance (ANOVA) test and Tukey’s *HSD* post hoc test. The change in expression of the examined genes was considered significant when *p* was less than 0.05. Statistical analyses were performed using the Statistical Package for Social Sciences (SPSS Inc., Chicago, IL, USA) version 23 for Windows. 

## 5. Conclusions

The most commonly used analgesics (ibuprofen, diclofenac, and paracetamol) had a significant effect on the mRNA expression of SARS-CoV-2 entry and arachidonic-acid-metabolizing genes in the mouse lung. These findings can explain, at least in part, the favorable use of paracetamol over NSAIDs in the management of pulmonary inflammation caused by viral infections, including COVID-19. 

## Figures and Tables

**Table 1:** The oligonucleotide sequence, amplicon size, and annealing temperature of mouse *ACE2, Ctsl, TMPRSS2, cox1, cox2, lox12, lox15, cyp4a12, cyp2j5, cyp2c29*, and *cyp3a11* genes.

Gene Name	Forward	Reverse	Size	Annealing Temp. (°C)	Reported in
*ACE2*	ATTCACCCAACACTTGAGCC	TGTCCATCGAGTCATAAGGGT	213	55	This study
*Cts l*	AGGAAAATGGAGGTCTGGACT	GCAACAGAAATAGGCCCCAC	205	58	This study
*TMPRSS2*	CGTTCCCGTATACTCCAGGT	CGTTCCCGTATACTCCAGGT	221	58	This study
*cyp3a11*	ACAAACAAGCAGGGATGGAC	GGTAGAGGAGCACCAAGCTG	250	53	[38]
*cyp2c29*	AGGAGTTTCCCAACCCAGAG	TTCTTTTGGGTGGACCAGAG	203	53	[38]
*cyp2j5*	GGGCCACTCCAGAAGTGTT	CTGGCTGGAGAAAGGATGAG	235	53	[38]
*cyp4a12*	GCCTTCATCACAACCCAACT	GGTATGGGGATTGGGACTCT	226	53	[39]
*alox12*	TGACGATGGAGACCGTGATG	GCT TTGGTCCTTGGGTCT GA	223	58	[39]
*alox15*	AAA GGCACTCTGTTTGAAGCG	CACCAAGTGTCCCCTCAG AAG	204	59	[38]
*cox2*	CCTCCATTGACCAGAGCAGA	GTGCTCGGCTTCCAGTATTG	247	58	[40]
*b-Actin*	CCCCTGAGGAGCACCGTGTG	ATGGCTGGGGTGTTGAAGGT	106	53	[41]

## Data Availability

The data are available from the corresponding authors.
